# Assessment of heart-substructures auto-contouring accuracy for application in heart-sparing radiotherapy for lung cancer

**DOI:** 10.1093/bjro/tzae006

**Published:** 2024-05-08

**Authors:** Tom Marchant, Gareth Price, Alan McWilliam, Edward Henderson, Dónal McSweeney, Marcel van Herk, Kathryn Banfill, Matthias Schmitt, Jennifer King, Claire Barker, Corinne Faivre-Finn

**Affiliations:** Christie Medical Physics & Engineering, The Christie NHS Foundation Trust, Manchester, M20 4BX, United Kingdom; Division of Cancer Sciences, The University of Manchester, Manchester, M13 9PL, United Kingdom; Division of Cancer Sciences, The University of Manchester, Manchester, M13 9PL, United Kingdom; Radiotherapy Related Research, The Christie NHS Foundation Trust, Manchester, M20 4BX, United Kingdom; Division of Cancer Sciences, The University of Manchester, Manchester, M13 9PL, United Kingdom; Radiotherapy Related Research, The Christie NHS Foundation Trust, Manchester, M20 4BX, United Kingdom; Division of Cancer Sciences, The University of Manchester, Manchester, M13 9PL, United Kingdom; Radiotherapy Related Research, The Christie NHS Foundation Trust, Manchester, M20 4BX, United Kingdom; Division of Cancer Sciences, The University of Manchester, Manchester, M13 9PL, United Kingdom; Radiotherapy Related Research, The Christie NHS Foundation Trust, Manchester, M20 4BX, United Kingdom; Division of Cancer Sciences, The University of Manchester, Manchester, M13 9PL, United Kingdom; Radiotherapy Related Research, The Christie NHS Foundation Trust, Manchester, M20 4BX, United Kingdom; Division of Cancer Sciences, The University of Manchester, Manchester, M13 9PL, United Kingdom; Department of Clinical Oncology, The Christie NHS Foundation Trust, Manchester, M20 4BX, United Kingdom; Division of Cardiovascular Sciences, The University of Manchester, Manchester, M13 9PL, United Kingdom; Department of Cardiology, Manchester University NHS Foundation Trust, Manchester, M13 9WL, United Kingdom; Department of Clinical Oncology, The Christie NHS Foundation Trust, Manchester, M20 4BX, United Kingdom; Department of Clinical Oncology, The Christie NHS Foundation Trust, Manchester, M20 4BX, United Kingdom; Division of Cancer Sciences, The University of Manchester, Manchester, M13 9PL, United Kingdom; Department of Clinical Oncology, The Christie NHS Foundation Trust, Manchester, M20 4BX, United Kingdom

**Keywords:** auto-contouring, artificial intelligence in RT, heart substructures, lung radiotherapy

## Abstract

**Objectives:**

We validated an auto-contouring algorithm for heart substructures in lung cancer patients, aiming to establish its accuracy and reliability for radiotherapy (RT) planning. We focus on contouring an amalgamated set of subregions in the base of the heart considered to be a new organ at risk, the cardiac avoidance area (CAA), to enable maximum dose limit implementation in lung RT planning.

**Methods:**

The study validates a deep-learning model specifically adapted for auto-contouring the CAA (which includes the right atrium, aortic valve root, and proximal segments of the left and right coronary arteries). Geometric, dosimetric, quantitative, and qualitative validation measures are reported. Comparison with manual contours, including assessment of interobserver variability, and robustness testing over 198 cases are also conducted.

**Results:**

Geometric validation shows that auto-contouring performance lies within the expected range of manual observer variability despite being slightly poorer than the average of manual observers (mean surface distance for CAA of 1.6 vs 1.2 mm, dice similarity coefficient of 0.86 vs 0.88). Dosimetric validation demonstrates consistency between plans optimized using auto-contours and manual contours. Robustness testing confirms acceptable contours in all cases, with 80% rated as “Good” and the remaining 20% as “Useful.”

**Conclusions:**

The auto-contouring algorithm for heart substructures in lung cancer patients demonstrates acceptable and comparable performance to human observers.

**Advances in knowledge:**

Accurate and reliable auto-contouring results for the CAA facilitate the implementation of a maximum dose limit to this region in lung RT planning, which has now been introduced in the routine setting at our institution.

## Introduction

Radiotherapy (RT) is a key component of curative-intent treatment for lung cancer. Recent studies have shown increasing evidence that excess mortality is related to irradiation of the heart.^1^^,^^2^ Specific substructures of the heart may be more sensitive to radiation, and data mining studies have revealed that higher radiation dose to the base of the heart is correlated with poorer survival.^3–6^ To monitor and reduce RT dose to the base of heart region, it is first necessary to contour the relevant substructures from the planning CT scan. However, contouring of heart substructures from RT-planning CT scans is challenging and time-consuming, which may limit the ability to reduce dose to this region. Heart substructures are often poorly visualized in RT-planning CT images due to respiration and cardiac motion, complex anatomy, and low contrast between structures. The development of methods to facilitate contouring of heart substructures, particularly the base of heart region, is therefore of clinical significance.

Automatic contouring methods have been investigated to address this issue. Multiple studies have reported auto-contouring systems to identify heart substructures from CT images, using a variety of approaches including: atlas methods based on deformable image registration,^7^^,^^8^ deep-learning methods based on convolutional neural networks (CNN),^9–14^ or hybrid methods.^15^ Studies vary in which substructures are included in the model. Most include the whole heart and the 4 main heart chambers (right and left atria and ventricles). Often great vessels are included (aorta, pulmonary artery, superior vena cava, inferior vena cava). Less frequently included are the coronary arteries (left main coronary artery, left anterior descending artery, left circumflex artery, and right coronary artery). The long, thin nature of the coronary artery structures and their often poor visualization on CT imaging make them particularly challenging to contour automatically. One solution to this problem is to define an expanded “high-risk” zone containing sensitive coronary arteries while being easier to contour when informed by surrogate landmarks.^16^

In this article, we report the validation of a deep-learning-based model for auto-contouring of a high-risk region at the base of the heart (subsequently referred to as cardiac avoidance area or CAA). This region was identified using image-based data mining techniques by McWilliam et al^4^ and encompasses the right atrium, aortic valve root, and proximal sections of the left and right coronary arteries. Rather than including all heart substructures in the auto-contouring model, we only contour those substructures which are to be included in the CAA. This reflects a difference in purpose to many studies where a large number of substructures are contoured individually for research into correlations between substructure dose and clinical outcomes. In the present study, we propose to incorporate a novel maximum dose limit to the CAA into the optimization of lung RT plans delivered at our institution, that is, to introduce the CAA as a new organ at risk (OAR) for lung RT planning. The aim of this validation study is to demonstrate auto-contouring performance suitable for this clinical implementation.

Following recommendations on validation and implementation of automated and artificial intelligence-based tools in RT,^17^^,^^18^ we used a range of validation metrics, including geometric and dosimetric, quantitative and qualitative. We also compared the quality of auto-contours to the expected range of manual contours due to interobserver variation. In contrast to previous studies, we assess this interobserver variation using both geometric and more clinically relevant dosimetric measures. Finally, we reviewed auto-contouring performance over 198 test cases to demonstrate the robustness of the model on a wide range of images.

## Methods

### Cardiac avoidance area

**Figure 1.**
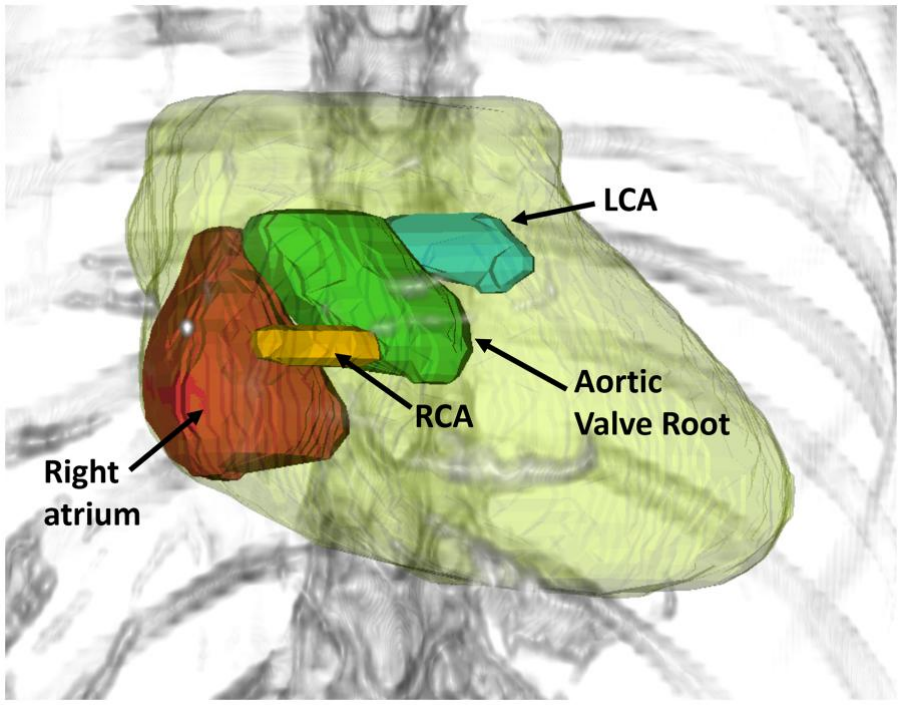
3D render of cardiac avoidance area showing right atrium (red), aortic valve root (green), proximal portions of left coronary artery (blue), and right coronary artery (orange). Whole heart outline is shown in yellow.

The CAA defined at our institution is based on the findings of McWilliam et al^4^ who identified, using image-based data mining techniques, a region at the base of the heart with excess radiosensitivity. The maximum dose to the base of the heart was found to be the most important factor associated with survival. After discussion with cardiologists and incorporating considerations of cardiac physiology,^19^ the final CAA includes the following structures located at the base of the heart: right atrium, the proximal portions of the left and right coronary arteries, and the aortic valve root. The aortic valve root structure includes the aortic valve and aortic root superiorly to the level of the coronary ostia or superior extent of the right atrium. The left and right coronary arteries are contoured with a standard width of 7 mm and only the first 2 cm from the coronary ostia is included. These short and wide artery structures are intended to include the portions coincident with the previously identified base of heart region and are also more easily contoured than if the full artery were included. Figure 1 shows a 3D render of the constituent substructures which are included in the CAA.

### Model validation

**Figure 2.**
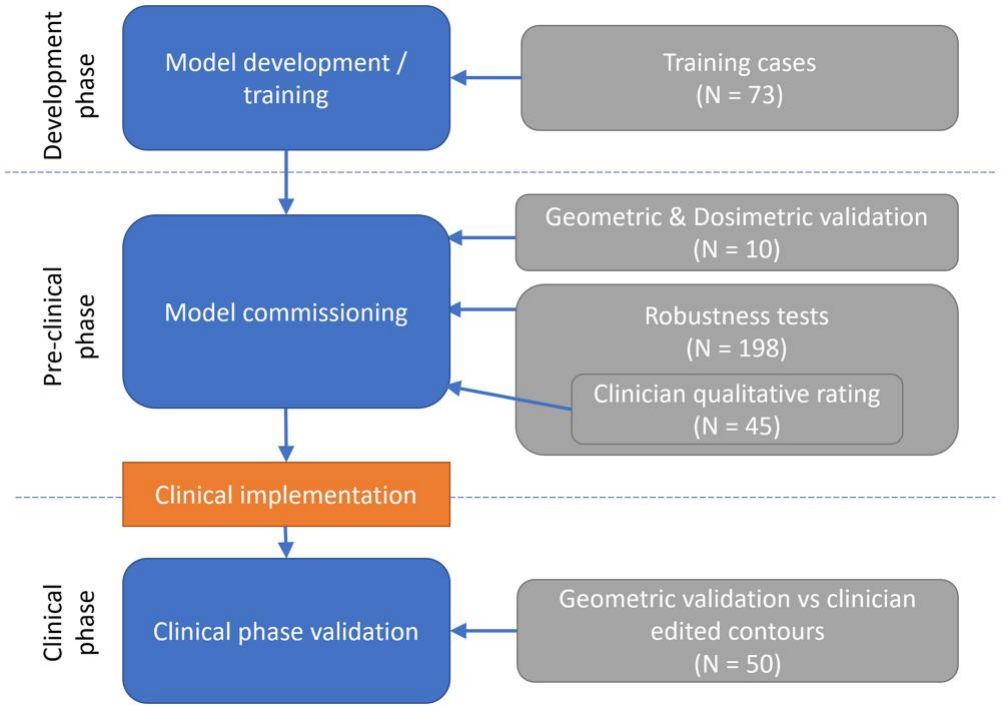
Flowchart of tests and data used at different stages of validation for heart substructure auto-contouring model.

An auto-contouring model for the CAA was developed based on a 3D CNN. The development and validation of the model were split into 3 phases (see Figure 2). An initial model development and training phase was followed by a preclinical model commissioning phase. The purpose of the commissioning process was to demonstrate acceptable performance for use of the auto-contouring model as part of a clinical workflow, using a variety of validation methods. Finally, clinical phase validation took place after implementation to verify performance in the clinical setting.

### CT image data

Auto-contouring methods were trained and assessed using CT image data from 331 patients treated with RT for lung cancer at a single centre (73 cases used for model training, 258 cases used for subsequent validation). Patient characteristics of cases used for model training and validation are shown in Table 1. Training and validation data were selected retrospectively from images acquired on 4 CT scanners (all Philips Brilliance Big Bore) using a 4D THORAX protocol (3-mm slice width, 512 × 512 matrix, 56- to 70-cm FOV, 120 kVp, 16 mm × 1.5 mm collimation, 0.059 pitch, 0.44-s rotation time, 47 mGy CTDIvol). For each case, the 4DCT average images only were used for model training and validation, and all patients received IV contrast unless clinically contraindicated. Each case consisted of multiple CT slice images covering the lungs of the patient, and the term “image” is subsequently used to refer to the full 3D scan, rather than individual slice images.

**Table 1.** Patient characteristics of cases used for training and validation of auto-contouring model.

	Training	Validation	All
Number of patients	73	258	331
**Patient sex**			
Male	30 (41.1%)	119 (46.1%)	149 (45.0%)
Female	43 (58.9%)	139 (53.9%)	182 (55.0%)
**Patient age (years)**			
Median (range)	71 (45-86)	74 (48-94)	73 (45-94)
**Primary disease**			
NSCLC	59 (80.8%)	212 (82.2%)	271 (81.9%)
SCLC	8 (11.0%)	11 (4.3%)	19 (5.7%)
Other/unknown	6 (8.2%)	35 (13.6%)	41 (12.4%)
**Disease stage**			
T1-3	51 (69.9%)	166 (64.3%)	217 (65.6%)
T4	9 (12.3%)	39 (15.1%)	48 (14.5%)
N0	31 (42.5%)	118 (45.7%)	149 (45.0%)
N+	29 (39.7%)	87 (33.7%)	116 (35.0%)
M0	64 (87.7%)	217 (84.1%)	281 (84.9%)
M1	0 (0.0%)	5 (1.9%)	5 (1.5%)
Unknown	13 (17.8%)	53 (20.5%)	66 (19.9%)
**Disease laterality**			
Left	29 (39.7%)	90 (34.9%)	119 (36.0%)
Right	30 (41.1%)	121 (46.9%)	151 (45.6%)
Bilateral/unknown	14 (19.2%)	47 (18.2%)	61 (18.4%)
**Performance status**			
ECOG 0	8 (11.0%)	15 (5.8%)	23 (6.9%)
ECOG 1	36 (49.3%)	108 (41.9%)	144 (43.5%)
ECOG 2	16 (21.9%)	79 (30.6%)	95 (28.7%)
ECOG 3	4 (5.5%)	21 (8.1%)	25 (7.6%)
Unknown	9 (12.3%)	35 (13.6%)	44 (13.3%)
**CT scan IV contrast**			
Yes	62 (84.9%)	224 (86.8%)	286 (86.4%)
No	11 (15.1%)	34 (13.2%)	45 (13.6%)

Abbreviations: NSCLC = Non-small cell lung cancer, SCLC = Small-cell lung cancer, ECOG = Eastern Cooperative Oncology Group, IV = Intravenous contrast.

### Auto-contouring with 3D CNN

An existing fast, efficient 3D CNN was adapted for cardiac substructures. The model is based on that previously used to segment abdominal organs,^20^^,^^21^ based on a 3D UNet design^22^ with added residual connections,^23^ and further modifications to reduce the model size and computational efficiency (see Supplementary Material for further details). The model uses the Pytorch deep-learning framework (v1.8.1). An initial step automatically identifies the heart location in the CT^24^ and crops the image around it to a size of 128 × 128 × 64 voxels.

Preprocessing is applied to the cropped image to remove high-density metal artefacts and to normalize the images into 2 separate contrast channels. Metal artefacts are identified using a threshold to create a mask of the high-density voxels. The high-density mask is then dilated and smoothed before being used to replace the included voxels with a soft-tissue density value. For image normalization, channel 1 uses a wider window to view general structures in the thoracic region (W1600, L-200), while channel 2 uses a narrower window to increase the contrast of the soft-tissue heart substructures (W200, L65). The segmentation CNN runs on the cropped image to infer masks representing each cardiac substructure, before these are postprocessed to remove small, disconnected regions via a morphological opening operation.^25^ Finally, the substructures are combined into the final CAA contour and padded back to the original image matrix size.

The deep-learning model was trained using 73 cases with manual contours for right atrium, aortic valve root, left coronary artery, and right coronary artery. These cases are assigned as either training (80%) or validation (20%), and the CNN was trained using a weighted multiclass soft Dice loss function^26^ and the Adam optimizer^27^ (loss function plots are shown in the Supplementary Material). The training process takes approximately 2 h using an NVidia GeForce RTX 3090 GPU. Once trained, the model is compiled using the Open Neural Network Exchange (ONNX),^28^ decreasing its size. During inference the CNN produces contours using the CPU alone, facilitating easy deployment of the model without specialist hardware. The final workflow uses the DICOM CT series as input and writes the segmented CAA and substructures contours as a DICOM RT-Structure.^29^

### Geometric validation

We evaluated the accuracy of our auto-contouring model against 3 sets of manual contours for 10 cases that were not included in the model training data (separate from the 73 cases used during model training). The 3 sets of manual contours were drawn by different observers (2 radiation oncologists and 1 physicist, all trained in contouring of the heart substructures involved).

We created a consensus contour from the 3 manual contours using the simultaneous truth and performance level estimation (STAPLE) method,^30^ which estimates a probabilistic ground truth and a performance level for each observer based on their agreement with others. We then compared all contours (manual and automatic) to the STAPLE contour using 3 metrics: mean surface distance (MSD), dice similarity coefficient (DSC), and Hausdorff distance 95th percentile (HD95).^31^ MSD measures the average distance between 2 contour surfaces in 3 dimensions,

(1) $$\mathrm{MSD}=\;\frac{1}{n_{S}+n_{S^{\prime }}}\left(\sum_{p=1}^{n_{S}}d\left(p,S^{\prime }\right)+\sum_{p^{\prime }=1}^{n_{S^{\prime }}}d(p^{\prime },S)\right),$$

where *S* and *S*′ are 2 contour surfaces consisting of points *p* and *p*′, and *d*(*p*, *S*′) is the minimum distance between point *p* (on surface *S*) and surface *S*′. MSD of zero indicates perfect agreement between contours, while higher values indicate increasing difference. DSC measures the spatial overlap between 2 segmentations, *A* and *B*, and is defined as $\mathrm{DSC}\left(A,B\right)=2(A\cap B)/(A+B)$, where ∩ is the intersection. DSC of one indicates complete overlap between segmentations, while zero indicates no overlap. HD95 measures the maximum distance between the contours, excluding the 5% of points which are furthest apart. This is a variation of the Hausdorff distance (HD) which measures the maximum distance between the contours at any point. HD95 is commonly used for the evaluation of segmentation accuracy in RT.^18^ It is a more robust measure than HD as it has lower sensitivity to statistical outlier points.^32^ HD95 of zero indicates the best agreement between contours, while higher values indicate increasing difference.

To investigate whether the auto-contours were consistent with the performance of the manual contours we classified each contour as either automatic or human generated. We then used R version 4.3.0^33^ and *lme4*^34^ to perform a linear mixed effects analysis of the relationship between MSD and contour type (automatic/human). The model included contour type as a fixed effect and patient as a random effect (since we expect random variations in contour quality between patients). *P*-values for the null hypothesis (that contour type does not affect MSD value) were generated from likelihood ratio tests of the full model against the same model without contour type as a fixed effect. This analysis was also repeated for DSC.

### Dosimetric validation

We further validated our auto-contouring model by generating lung RT plans using the manual and automatic contours for the CAA. This was done to assess the clinical impact of using automatic contours for RT planning. All plans were created with the Philips Pinnacle treatment planning system (v16) using a dual arc VMAT technique to deliver 55 Gy in 20 fractions to the Planning Target Volume (PTV). A maximum dose objective of 19.5 Gy was set for the CAA during optimization (see Supplementary Material for further details of RT planning objectives). To ensure consistent plan quality, all plans were generated automatically using a scripted process without manual intervention.

For each of the 10 cases, we created 5 plans: 1 optimized using each of the 3 manual CAA contours, 1 using the STAPLE contour, and 1 using the automatic contour. We compared a range of target and OAR dose statistics between these plans: PTV 1-cc min, PTV D95%, PTV 1-cc max, PTV mean, spinal cord max, spinal cord 1-cc max, lungs-PTV V5Gy, lungs-PTV V10Gy, lungs-PTV V20Gy, lungs-Internal Target Volume (ITV) mean, heart V30Gy, heart V40Gy, CAA max, CAA 1-cc max, oesophagus mean, and oesophagus V55Gy.

For all plans, we calculated the CAA dose metrics using the STAPLE contour, regardless of which contour was used for plan optimization, as this was the best estimate of the true CAA contour. For each dose metric, the consistency of the plan generated using automatic contours with the plans generated using manual contours was tested using the same linear mixed effects analysis as used for the geometric validation.

### Robustness testing and qualitative scoring

Robustness of the contouring tool was investigated by applying to a larger test set of 198 CT scans from the same centre. The aim was to investigate the consistency of the proposed contouring algorithm and to reveal any rarer cases of contouring failure which may not be apparent with the smaller test set used for the geometric and dosimetric validations. Auto-contours for all 198 cases were reviewed visually on all slices to check that contours were produced and identify any gross failures (ie, no overlap with actual CAA). Additionally, 45 auto-contours (randomly selected from the 198 cases used for robustness testing) were rated by an expert clinician (J.K.) using a 1-5 Likert scale, as shown in Table 2.

**Table 2.** Qualitative scoring scale used to rate auto-contour quality.

1	No output/software crash
2	Not useful/needs contouring from scratch
3	Useful/significant changes needed
4	Good/minor edits only
5	Very good/no changes needed

### Assessment of clinician-edited contours

Following the clinical implementation of the CAA auto-contouring software, the quality of auto-contours for the first 50 patients was assessed. All auto-contours were checked by a clinician and edited where necessary to produce a CAA contour that was suitable to use for plan optimization. The clinician-accepted contours were then compared to the original auto-contours in terms of MSD and DSC to evaluate the degree of editing that was required.

## Results

### Auto-contouring with 3D CNN

**Figure 3.**
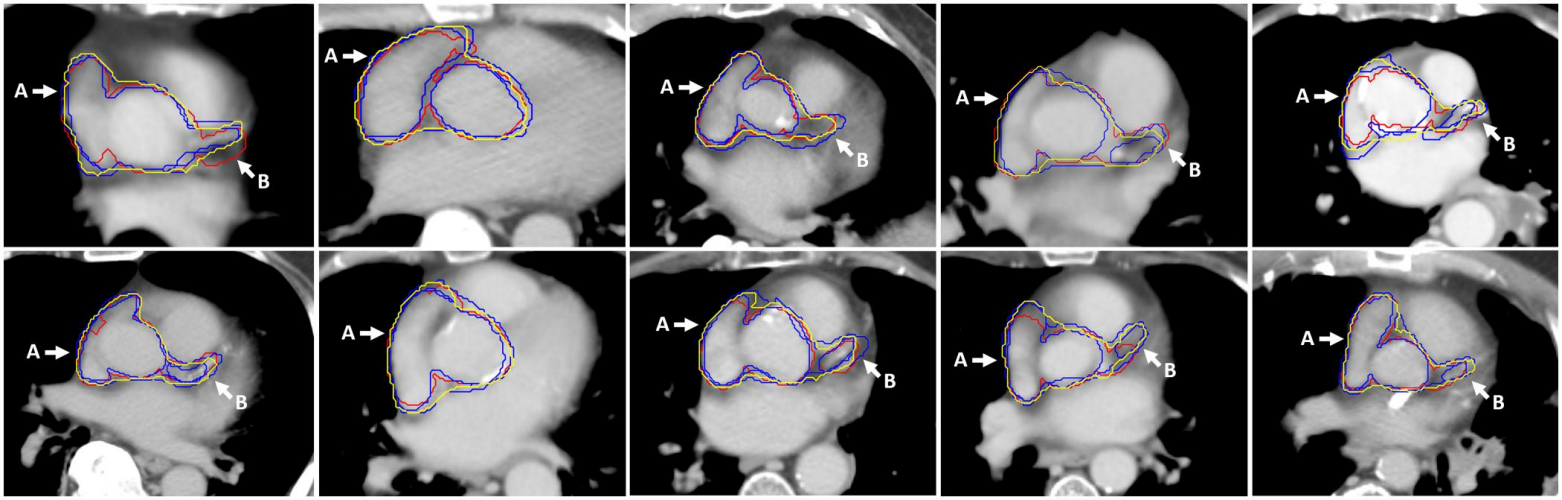
Comparison of CAA auto-contours (red), manual contours (blue) and STAPLE consensus contours (yellow). Arrows A indicate border between right atrium and lung tissue with generally good agreement between manual and automatic contours. Arrows B indicate left coronary artery with greater variation between contours. CAA = cardiac avoidance area; STAPLE = simultaneous truth and performance level estimation.

Figure 3 illustrates example CAA contours for the 10 validation patients produced by the 3D CNN (red), along with manual contours from 3 different observers (blue) and the consensus manual contour (yellow). Variation between the different manual contours, the automatic contour, and the consensus contour can be observed. Contour variation tends to be smaller at the border between right atrium and lung tissues (indicated by arrows A in Figure 3) and greater around the left coronary artery (arrows B in Figure 3). CNN inference produced the cardiac substructure masks in approximately 1 s per case (running on an Intel Xeon 6134 CPU), although full processing took 30-60 s per case (including data in/output, cropping, and postprocessing).

### Geometric validation

**Figure 4.**
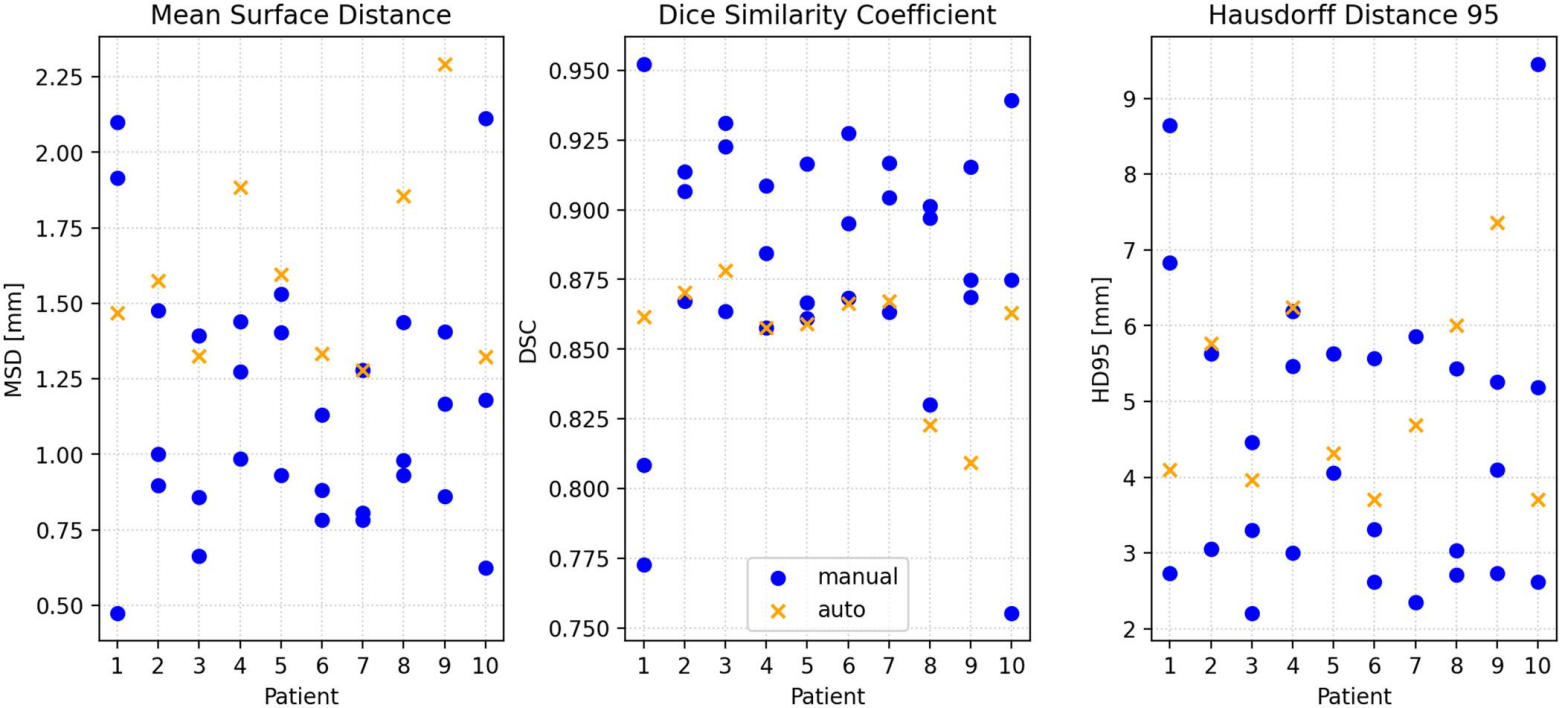
Comparison of MSD and DSC between manual and automatic contours relative to consensus STAPLE contour for 10 patients. DSC = dice similarity coefficient; MSD = mean surface distance; STAPLE = simultaneous truth and performance level estimation.

Figure 4 shows the results of geometric validation metrics MSD, DSC, and HD95 for manual contours (blue dots) and auto-contours (orange crosses) compared to the STAPLE consensus contour.

Table 3 shows the results of the geometric validation metrics for auto-contours and manual contours. For each metric (MSD, DSC, and HD95), the auto-contour mean value over the 10 patients is shown, along with the mean value for manual contours (averaged first over the 3 observers for each patient and then over the 10 patients). Also shown is the standard deviation between the manual observers (calculated separately for each patient and then averaged over patients), and the *P*-value for the metric not being dependent on contour type (automatic/human) calculated from the mixed effects model.

**Table 3.** Comparison of auto vs manual contours geometric validation metrics.

Metric	Auto contours mean	Manual contours mean	Manual contours, SD (mean over patients)	Mixed effects model *P*-value
MSD	1.593	1.157	0.389	.004
DSC	0.856	0.882	0.043	.076
HD95	4.985	4.415	1.799	.364

Abbreviations: DSC = dice similarity coefficient; HD95 = Hausdorff distance 95th percentile; MSD = mean surface distance.

For each patient, a range of values for the manual contours is observed. For DSC, the auto-contour value is generally around the lower end of this range (or upper end of the range for MSD and HD95). This indicates that the auto-contour performance is slightly poorer than the manual observers on average, although it is consistent with the range of performance expected from manual observers. This observation is reflected by the mean values in Table 3 where the auto-contour mean DSC (0.86) is slightly poorer than the manual contour value (0.88). The mixed effects model analysis indicates that DSC and HD95 are not significantly correlated with contour type (automatic vs manual), although for MSD the effect was significant.

### Dosimetric validation

**Figure 5.**
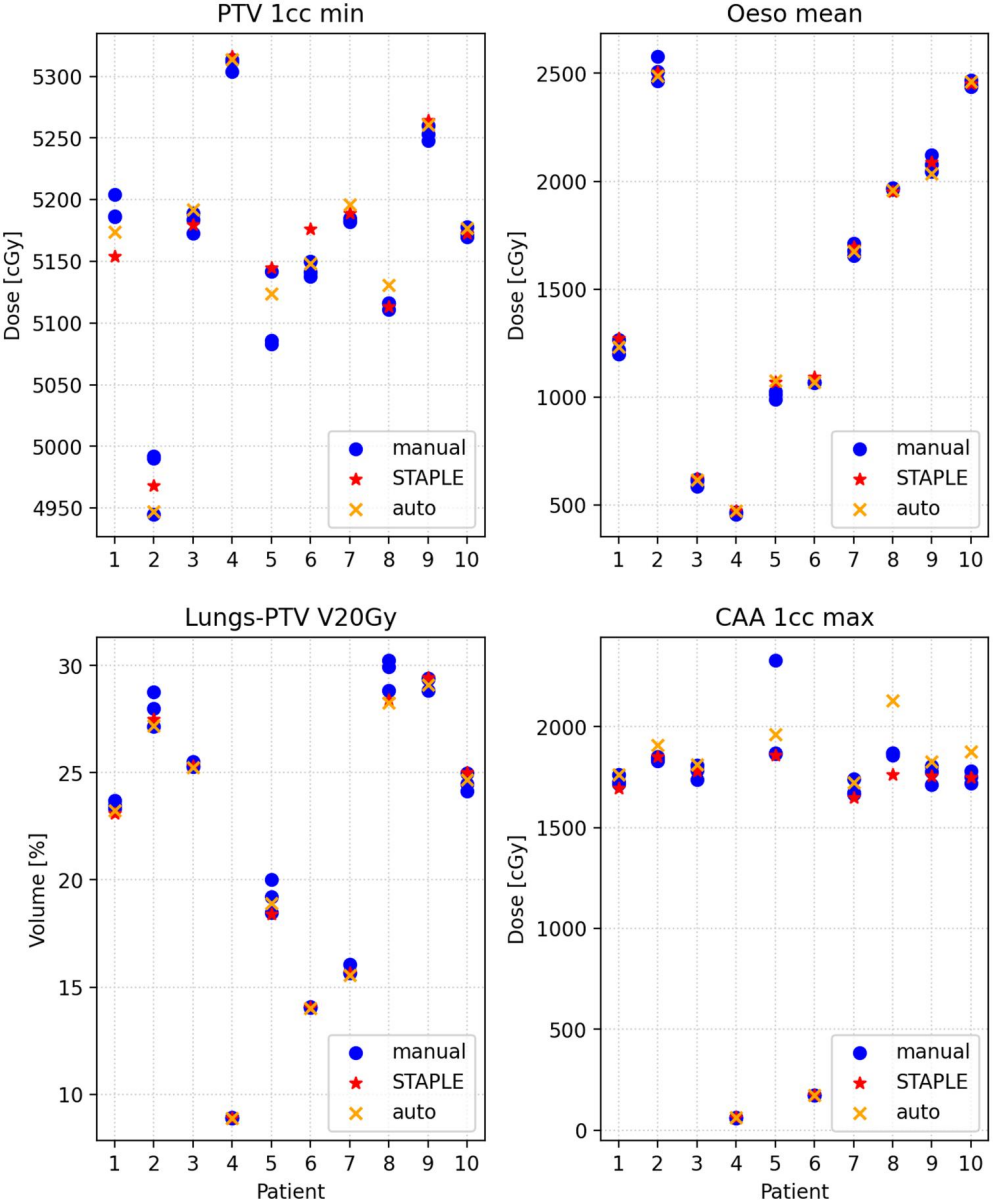
Comparison of dose statistics between plans optimized using automatic and manual CAA contours. CAA = cardiac avoidance area.

Figure 5 shows dose metrics for lung RT plans with CAA sparing generated using automatic, manual, and STAPLE CAA contours (additional dose metrics are shown in the Supplementary Material). The blue dots illustrate the range of values achieved for plans optimized using the different manual contours for the CAA. The orange cross indicates the plan optimized using the CAA auto-contour, which in the majority of cases is within the range of the manual plans (blue dots).

Table 4 shows results of the dosimetric validation metrics compared between plans optimized using auto-contours and plans optimized using manual contours. For each dose metric, the auto-contour mean value over the 10 patients is shown, along with the mean value for manual contour plans (averaged first over the 3 observers for each patient, and then over the 10 patients). Also shown is the standard deviation between the manual observer plans (calculated separately for each patient and then averaged over patients), which indicates how much variation in each dose metric occurs due to interobserver variation in the manual CAA contours. and the final column shows the *P*-value for the metric not being dependent on the contour type (automatic/manual) calculated from the mixed effects model. Only the CAA maximum dose was significantly dependent on contour type. Significance testing was done using a significance level of α = 0.05, without correction for multiple comparisons. Note that correcting for multiple comparisons via either the Bonferroni or Holm methods^35^ would result in none of the dose metrics being significant. Dose-volume histograms of the CAA for plans optimized using automatic and manual CAA contours are shown in the Supplementary Material.

**Table 4.** Comparison of auto vs manual contours for dosimetric validation metrics.

Metric	Auto contours mean	Manual contours mean	Manual contours SD	Mixed effects model *P*-value
PTV_IMRT 1-cc min	5166.4	5163.1	10.4	.539
PTV_IMRT D95	5327.3	5327.3	7.3	1.000
PTV_IMRT 1-cc max	5639.7	5642.8	10.9	.521
PTV_IMRT mean	5469.2	5470.4	1.7	.239
ITV 1-cc min	5404.8	5402.8	7.7	.590
ITV D95	5442.1	5440.2	3.6	.443
ITV 1-cc max	5583.3	5586.7	7.9	.325
ITV mean	5499.9	5500.1	0.5	.267
SC max	2941.5	2961.3	46.7	.577
SC 1-cc max	2690.5	2709.1	62.5	.645
SC + 0.5 cm max	3300.2	3324.2	36.7	.284
SC + 0.5 cm 1-cc max	2991.5	3011.2	46.7	.515
Lungs-PTV V5Gy	61.3	61.3	0.3	.930
Lungs-PTV V10Gy	42.9	43.0	0.4	.805
Lungs-PTV V20Gy	21.5	21.8	0.4	.072
Lungs-ITV mean	1323.7	1328.3	5.1	.357
Heart V30Gy	10.7	10.5	0.3	.181
Heart V40Gy	6.3	6.1	0.1	.076
CAA max	2115.1	1754.4	147.6	.012
CAA 1-cc max	1523.8	1468.4	46.7	.087
Oeso mean	1508.3	1507.5	22.0	.941
Oeso V55Gy	3.1	3.1	0.8	.964

Abbreviations: CAA = Cardiac Avoidance Area, PTV = Planning Target Volume, IMRT = Intensity Modulated Radiotherapy, D95 = Dose covering 95% of structure volume, ITV = Internal Target Volume, SC = Spinal Cord, VXGy = Volume of organ receiving greater than X Gray, Oeso = Oesophagus.

### Robustness testing

Auto-contouring runs successfully with no gross failures for all 198 tested cases. Of 45 auto-contours rated by an expert clinician, 80% were rated as “Good” (minimal editing required), with the remaining 20% of cases rated as “Useful.”

### Assessment of clinician-edited contours

**Figure 6.**
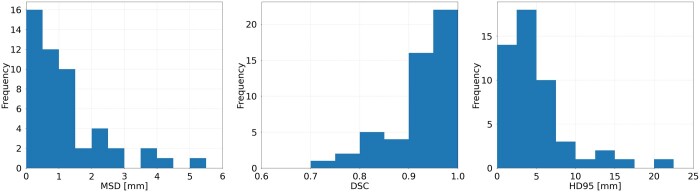
MSD and DSC values for between original auto-contour and clinician-edited contour for 50 cases. DSC = dice similarity coefficient; MSD = mean surface distance.

Figure 6 shows histograms of the MSD and DSC values between original auto-contours and clinician-accepted contours for 50 lung cancer cases which were planned with sparing of the CAA. The mean MSD value is 1.2 mm, the mean DSC value is 0.93, and the mean HD95 value is 4.6 mm.

## Discussion

The deep-learning auto-contouring model for the CAA was validated using a range of metrics. Our results show that the auto-contours were acceptable, and consistent with the performance of human observers for most metrics. Auto-contouring performance for the CAA cannot be directly compared to other published heart substructure auto-contouring models, as these do not include the combined CAA as structure. However, the mean DSC of 0.86 for the combined CAA structure used in our model is consistent with the performance of other reported models for the main heart chambers, which are in the range 0.75-87,^15^ 0.78,^7^ 0.70-0.79,^8^ 0.89,^9^ 0.81-0.93,^10^ 0.87-0.92,^12^ 0.76-0.88,^13^ and 0.82-0.88.^14^ It is worth noting that the assessment of clinician-edited contours after clinical implementation showed better results than the initial testing (DSC of 0.93 vs 0.86, and MSD of 1.2 vs 1.6 mm). This is to be expected as the clinician-edited contours in the clinical implementation use the auto-contour as a starting point whereas in the initial testing, clinicians contoured independent of the auto-contour. This illustrates that results in the clinical setting are not always the same as those from the commissioning (preimplementation) phase. It is therefore important to repeat validation tests after implementation of an Artificial Intelligence (AI)-based tool, to ensure that acceptable performance is maintained.^36^

We also validated our auto-contouring model against the expected interobserver variation between manual observers, finding a mean DSC of 0.88 between manual observers for the CAA. This is similar to the value for auto-contours generated using our model (of 0.86). DSC values for all contours were calculated in comparison to the STAPLE consensus contour generated from the 3 manual observer contours. This will tend to reduce the apparent error for the manual contours as the STAPLE contour will be biased towards them. Although the structure studied here is not directly comparable, the result is similar to the degree of interobserver variation seen for the larger heart substructures by Milo et al^37^ where median DSC 0.78-0.96 was found for whole heart and cardiac chambers, and by Zhou et al,^8^ where DSC 0.84-0.90 was reported for main heart chambers.

We analysed dose statistics of lung RT plans optimized to reduce dose to the CAA, comparing plans based on auto-contours to those based on manual contours. A distinguishing feature of this study is the consideration of interobserver variation in the dose-based validation of the contouring model. We found that plans based on auto-contours were consistent with plans optimized using manual contours for all dose metrics except for CAA maximum dose, which tended to be higher for plans optimized using automatic CAA contours. However, the difference for this metric became insignificant after adjusting for multiple comparisons. It is not surprising that this dose metric was the most sensitive to small differences in the CAA contour, since the plans were specifically optimized to reduce dose to this structure. The resulting plans often have a steep gradient in the dose distribution adjacent to the CAA. Small differences in the CAA contours can therefore lead to a large change in the maximum dose to that structure. However, the 1-cc max dose to CAA metric was found to be consistent between automatic and manual contours, indicating that the largest differences between the plans were limited to a small volume.

Our auto-contouring robustness test with 198 cases and qualitative scoring with 45 cases showed that the software produces contours free of gross errors in all cases and that 80% of cases were rated as “Good,” only requiring minimal editing for clinical use. The remaining 20% of contours were rated as “Useful,” with no contours rated as “Not useful.” Other studies to report qualitative scoring of heart substructure auto-contours include Garrett Fernandes et al^12^ where heart chamber contours were rated clinically acceptable in 96%-100% of 99 cases; Walls et al^11^ rated 20 patients, finding all heart chamber auto-contours to be either Good or Acceptable; Haq et al^10^ rated 25 cases, finding that 85% were acceptable for clinical use with no adjustments. In a large cohort of 1429 cases, Bruns et al^9^ found 83%-96% acceptable auto-contour quality for the main heart chambers, although only selected slices were reviewed rather than the full contour.

Following the validation studies reported in this article, the CAA auto-contouring model has been implemented in the routine clinical setting. Since April 2023, curative-intent, non-SABR lung cancer patients at our institution are planned with optimization objectives to reduce dose to the CAA, which represents a new OAR. The use of the auto-contouring tool has facilitated the implementation of CAA-sparing RT planning in all patients with stage 1-3 Non-Small Cell Lung Cancer (NSCLC) treated with non-SABR curative-intent RT at our institution. Indeed, the majority of clinical oncologists on the lung team were not familiar with the contouring of this region and have limited time due to large workload. Deep-learning-based auto-contours of the CAA are generated and imported to the RT treatment planning system. The contours are then checked (and if necessary edited) by a radiation oncologist before being used for plan optimization.^38^ Any changes in clinical outcomes resulting from limiting dose to the CAA will be analysed prospectively using rapid-learning methodology and real-world data as part of the RAPID-RT project.^39^

## Conclusion

In this study, we have developed a CNN-based tool that can automatically contour the CAA for lung RT patients. We have validated the auto-contouring performance using various methods, such as geometric and dosimetric measures, and compared it with the interobserver variation of manual contours. Our results show that the auto-contours and the plans optimized using them are consistent with the manual contours and the plans optimized using them. In addition, the auto-contouring tool is robust, and performance monitoring since clinical implementation has shown that only a small degree of manual contour editing is required. The development of the auto-contouring system facilitates the clinical implementation of dose reduction to the CAA, which may improve patient outcomes by reducing cardiac toxicity.

## Supplementary Material

tzae006_Supplementary_Data
