# Supplementary material for: Assessment of heart-substructures auto-contouring accuracy for application in heart-sparing radiotherapy for lung cancer
Source: BJR Open. 2024 May 8;6(1):tzae006. doi: 10.1093/bjro/tzae006 (PMC11087931; doi:10.1093/bjro/tzae006)
Supplement: tzae006_Supplementary_Data [file tzae006_supplementary_data.zip › Supplementary-material.pdf]

Supplementary material

3D CNN architecture for auto-contouring of Cardiac Avoidance Area

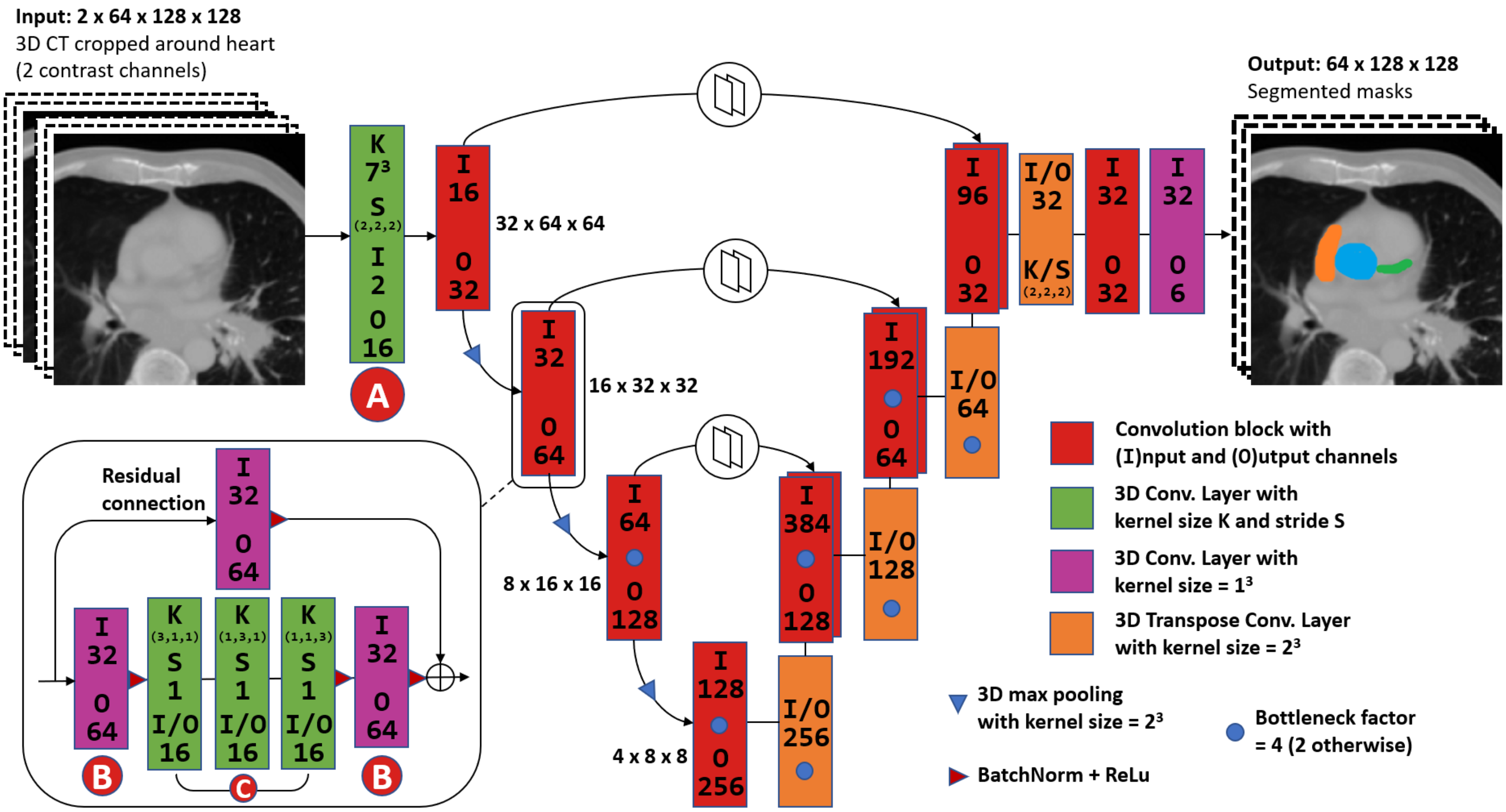

Figure S1: 3D CNN architecture for heart substructures segmentation (adapted from Henderson[1]).

A diagram of our custom 3D CNN architecture. Our network is influenced by a standard 3D UNet [2] with added ResNet-like residual connections[3]. In order to reduce the model size and computational load we introduce three components: a YOLO-inspired[4] 7x7x7 input convolution with stride 2 to quickly reduce the size of the input image whilst preserving a large field of view (see A); bottleneck structures to reduce the number of kernels required (B); and asymmetric factorisation of convolution layers (C).

1. Henderson EGA, McSweeney DM, Green AF. COBRA: Cpu-Only aBdominal oRgan segmentAtion. Published online July 21, 2022. doi:10.48550/arxiv.2207.10446

2. Çiçek Ö, Abdulkadir A, Lienkamp SS, Brox T, Ronneberger O. 3D U-net: Learning dense volumetric segmentation from sparse annotation. *Lecture Notes in Computer Science (including subseries Lecture Notes in Artificial Intelligence and Lecture Notes in Bioinformatics)*. 2016;9901 LNCS:424-432. doi:10.1007/978-3-319-46723-8_49

3. He K, Zhang X, Ren S, Sun J. Deep residual learning for image recognition. *Proceedings of the IEEE Computer Society Conference on Computer Vision and Pattern Recognition*. 2016;2016-December:770-778. doi:10.1109/CVPR.2016.90

4. Redmon J, Divvala S, Girshick R, Farhadi A. You Only Look Once: Unified, Real-Time Object Detection. Published online 2016. doi:10.1109/CVPR.2016.91

CNN Training details

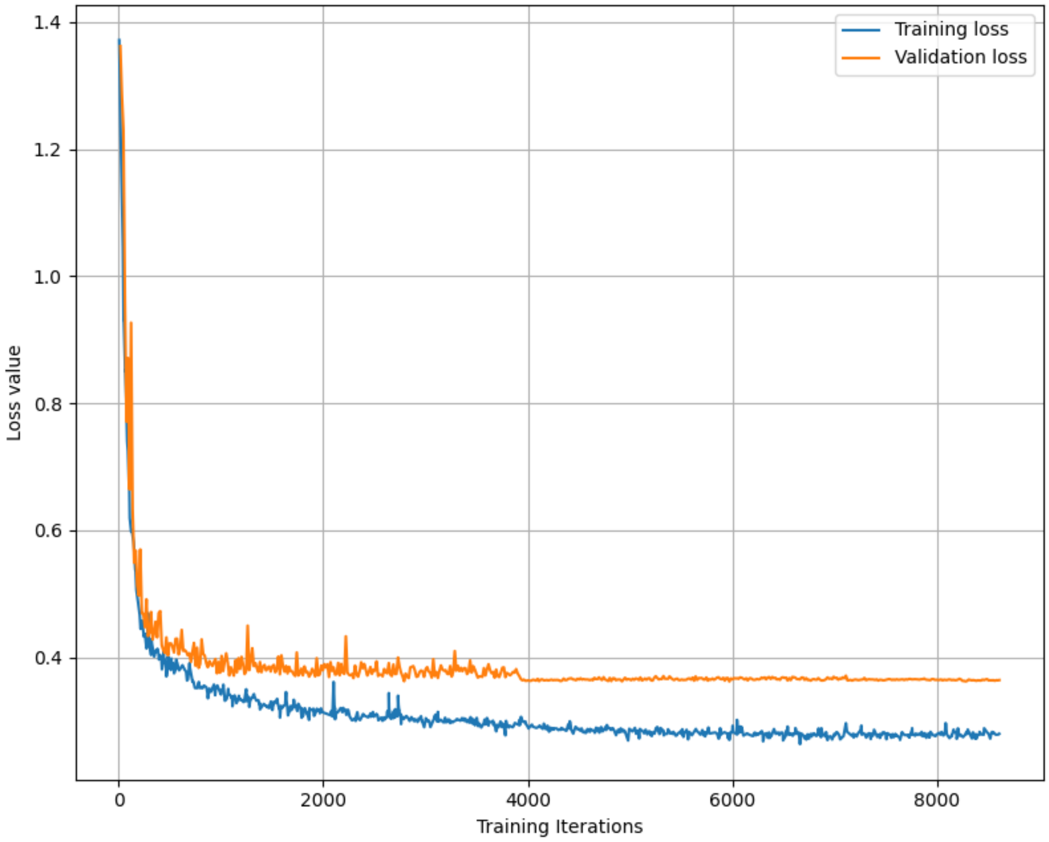

Figure S2: Loss function of contouring model during training process for training and validation data set.

Lung VMAT radiotherapy planning objectives

ROI	*Type*	*Target (cGy)*	*% Volume*	*Weight*
Body – (PTV+5cm)	Max Dose	3000	-	10
ITV	Min Dose	5000	-	50
ITV	Uniform Dose	5000	-	12
Heart – (PTV+3cm)	Max DVH	3000	30	10
Inner Rind	Max Dose	5250	-	10
Lungs – (PTV+5cm)	Max Dose	3000	-	10
Lungs-PTV	Max DVH	2000	30	10
Lungs-PTV	Max DVH	1000	60	2
PTV_IMRT	Min DVH	4850	99	50
PTV_IMRT	Min DVH	4900	90	60
PTV_IMRT	Min Dose	4800	-	50
PTV_IMRT	Max Dose	5200	-	30
PTV_Lim1	Max Dose	4650	-	20
PTV_Lim2	Max Dose	3250	-	40
SC+0.5cm	Max Dose	4000	-	50
Oesophagus – (PTV+0.5cm)	Max DVH	5000	35	1
Oesophagus – (PTV+0.5cm)	Max EUD	3400	-	1
Cardiac Avoidance Area – (PTV+1.5cm)	Max Dose	1950	-	25

Inner rind is a rind surrounding the PTV with width of 1cm (in lung tissue) or 0.5cm (elsewhere).
PTV_Lim1 is a 1cm rind surrounding Inner rind.
PTV_Lim2 is the region of body outside of PTV_Lim1.

Comparison of Lung VMAT dose distributions between manual and automatic contours

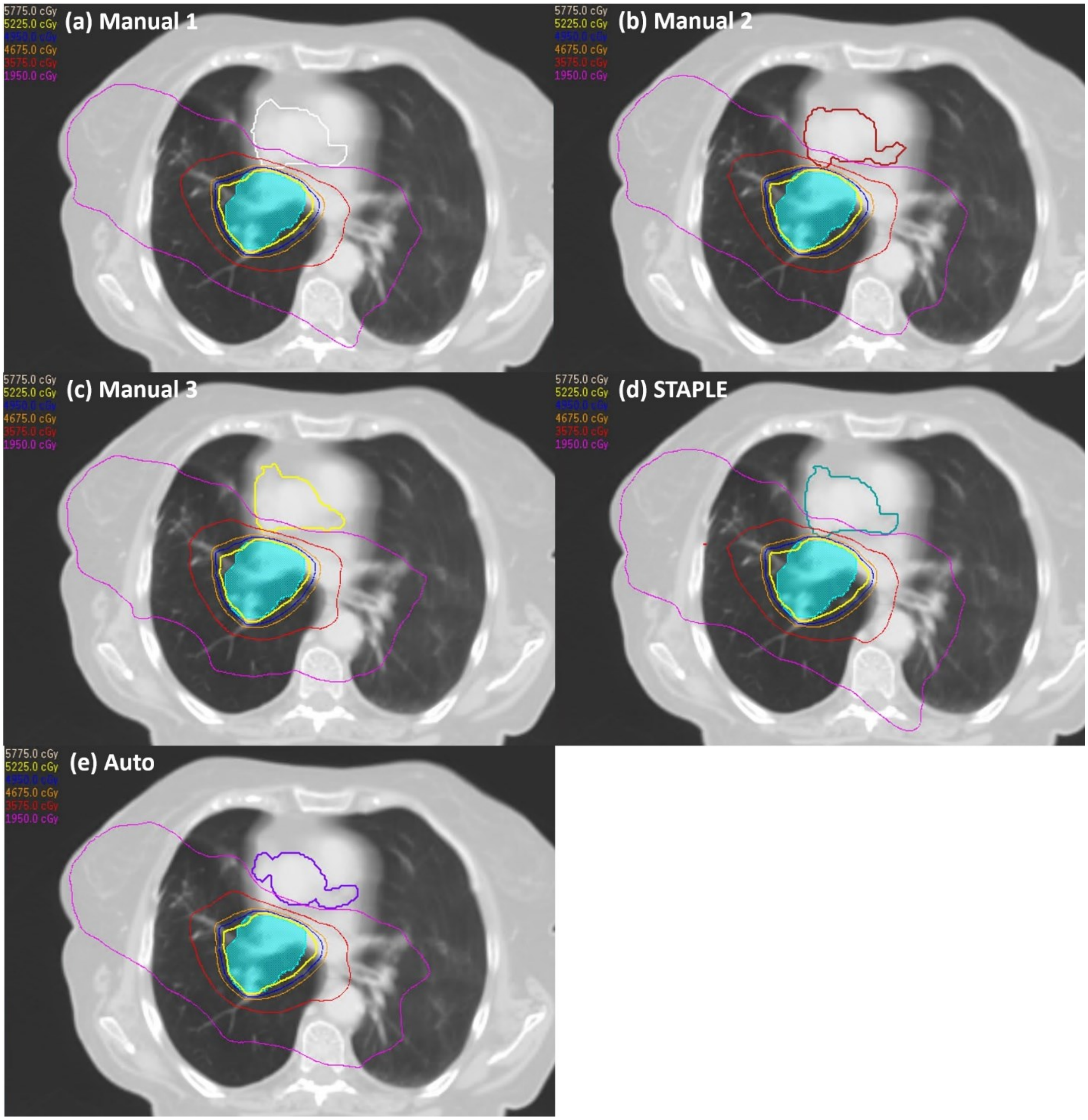

Figure S3: Comparison of lung VMAT dose distributions for an example patient optimized with manual or automatic CAA contours. (a-c) Plans optimized with three manual observer CAA contours (white, dark red and yellow contours). (d) Plan optimized with STAPLE consensus CAA contour (teal contour). (e) Plan optimized using automatic CAA contour (purple contour).

Results: Dose metrics for plans optimised using manual and automatic CAA contours

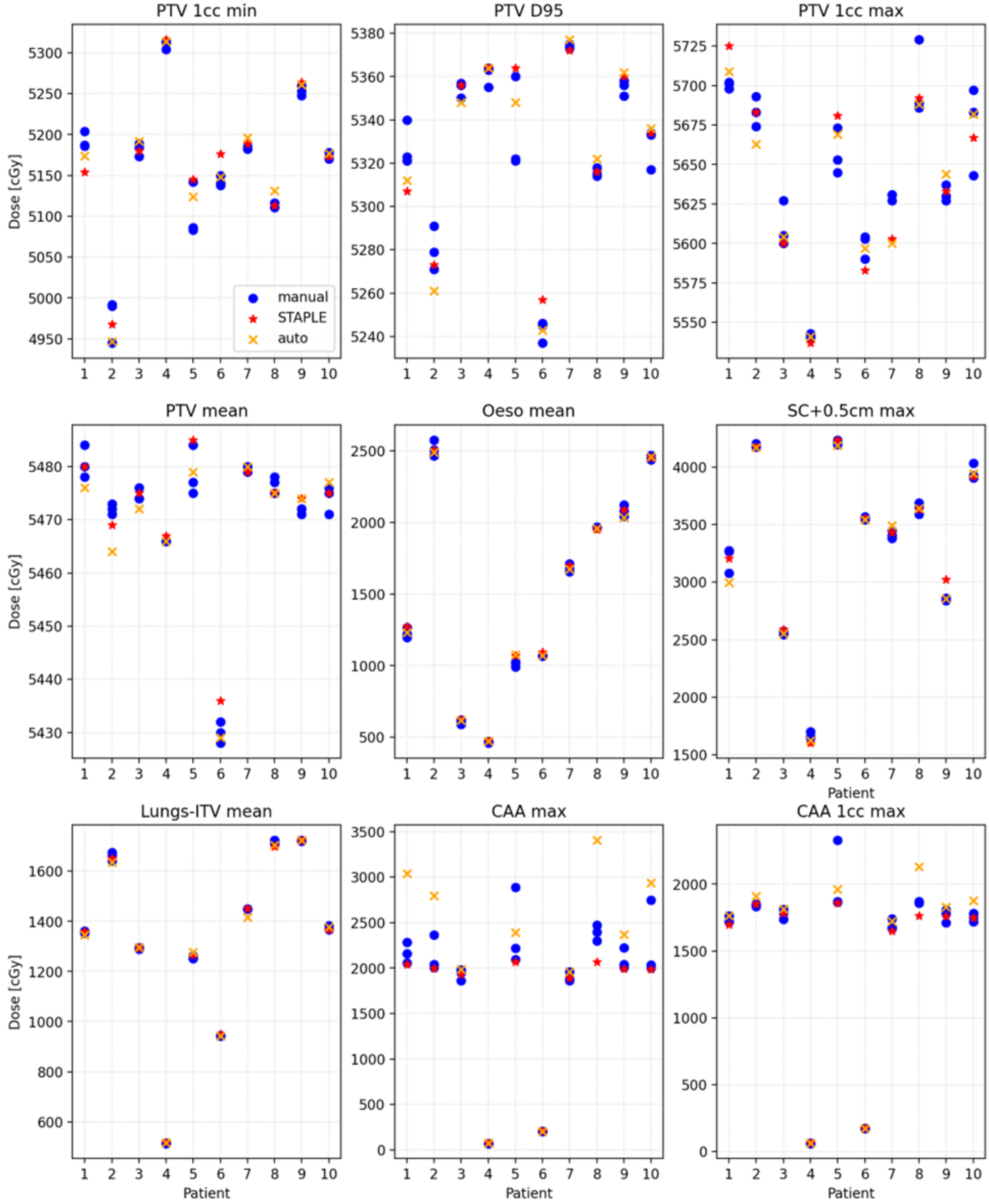

Figure S4: Comparison of dose statistics between plans optimised using automatic and manual CAA contours.

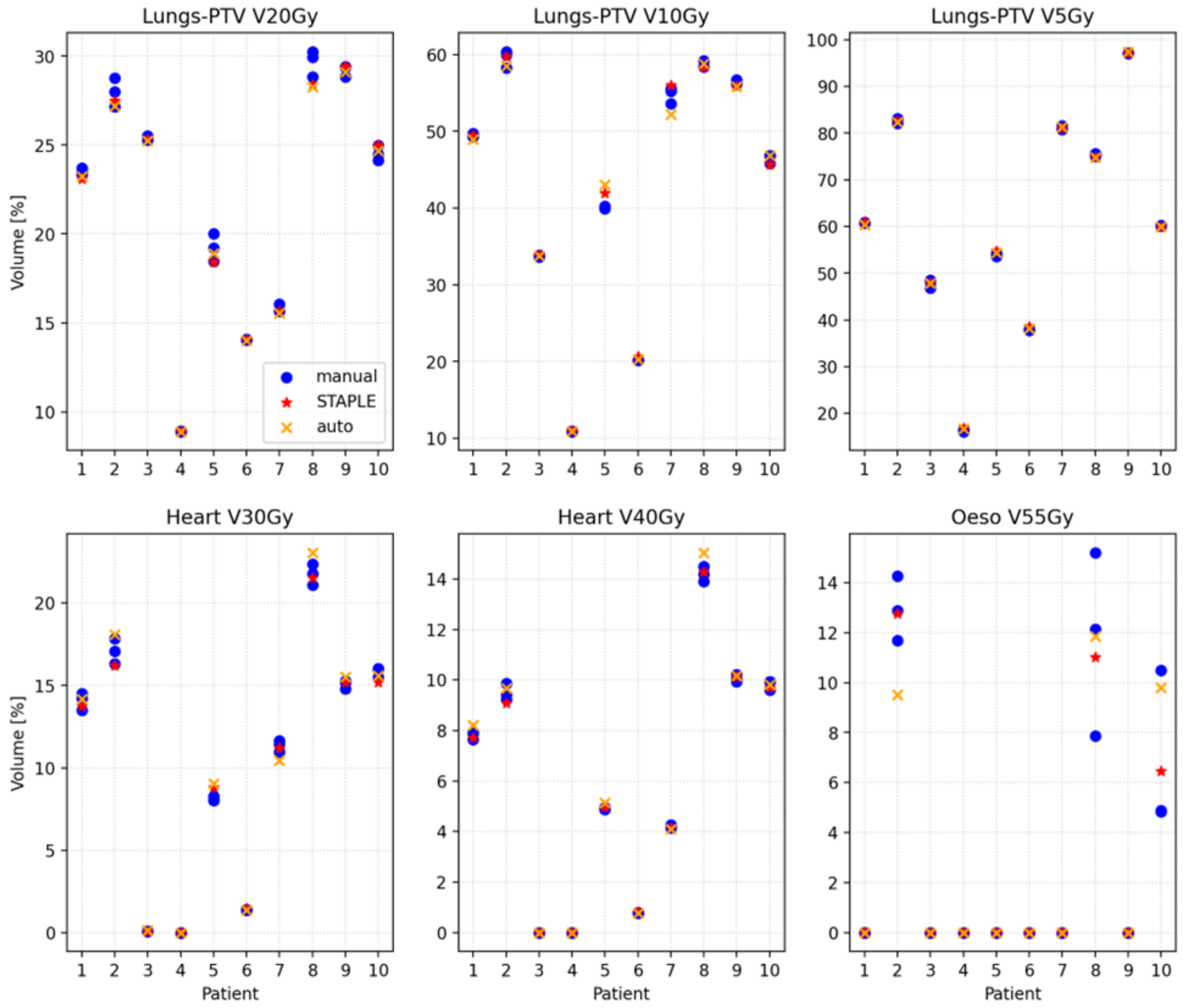

Figure S5: *Comparison of dose volume statistics between plans optimised using automatic and manual CAA contours.*

Cardiac Avoidance Area Dose Volume Histograms for plans optimized using manual vs automatic contours

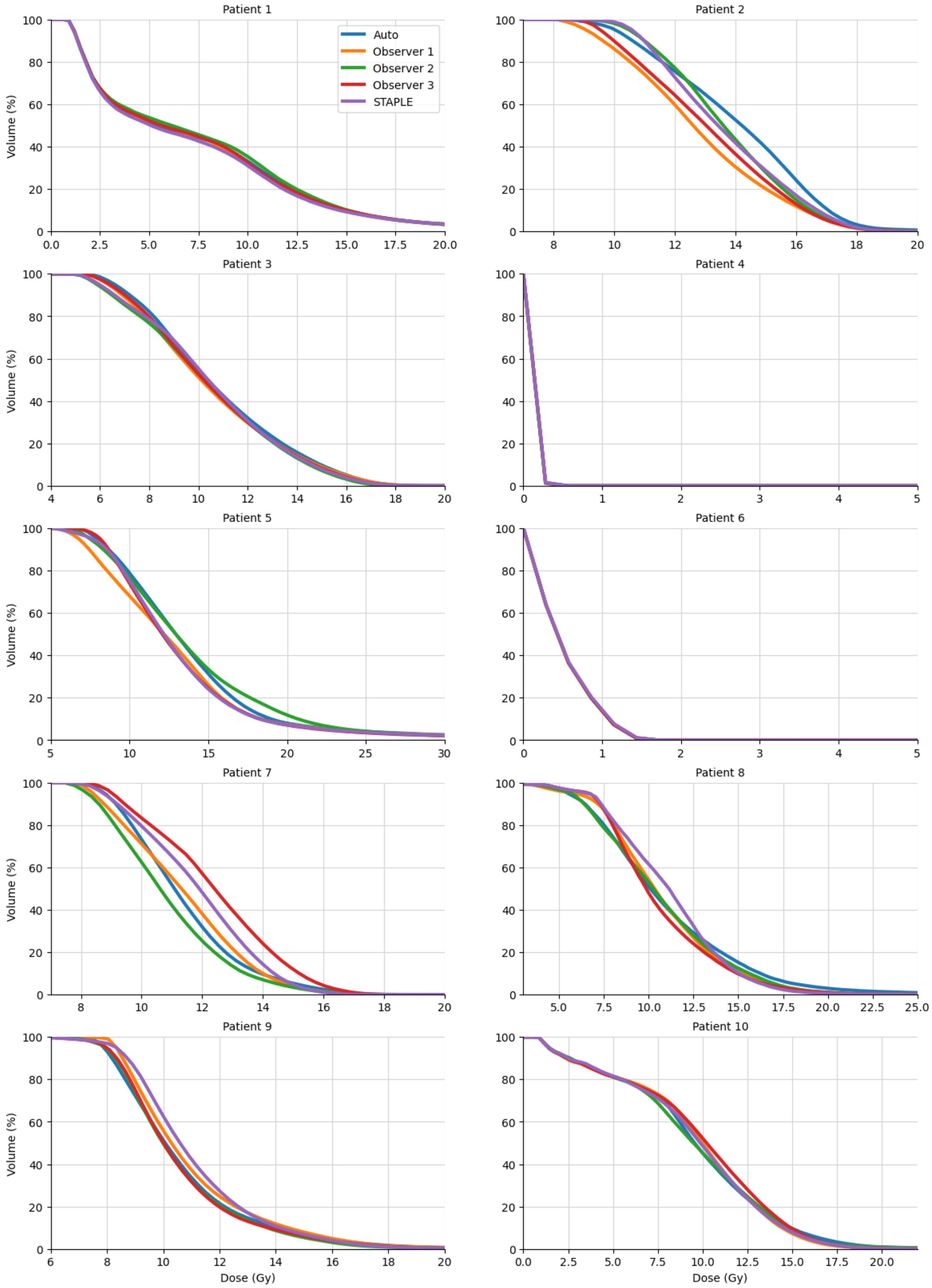

Figure S6: CAA DVH plots for plans optimized using manual and automatic CAA contours. DVHs are calculated using the STAPLE CAA contour in all cases. Note CAA maximum dose for patient 4 is near zero due to target volume out of plane with CAA.
